# Optimism is associated with respiratory symptoms and functional status in chronic obstructive pulmonary disease

**DOI:** 10.1186/s12931-021-01922-6

**Published:** 2022-01-29

**Authors:** Hyeon-Kyoung Koo, Karin F. Hoth, Barry J. Make, Elizabeth A. Regan, James D. Crapo, Edwin K. Silverman, Dawn L. DeMeo

**Affiliations:** 1 Channing Division of Network Medicine, Brigham and Women’s Hospital, 181 Longwood Avenue, Boston, MA 02115 USA; 2 Division of Pulmonary and Critical Care Medicine, Ilsan Paik Hospital, Inje University College of Medicine, Ilsan, Republic of Korea; 3 Department of Psychiatry, University of Iowa Carver College of Medicine, Iowa, USA; 4 National Jewish Health, Denver, CO USA; 5 Division of Pulmonary and Critical Care Medicine, Brigham and Women’s Hospital, Boston, MA USA

**Keywords:** Optimism, COPD, Exacerbation, Dyspnea, Quality of life

## Abstract

**Background:**

Optimism is the general belief that good things will occur in the future; optimism is modifiable by cognitive behavioral therapy (CBT). Previous studies have associated higher optimism with improved health outcomes and lower all-cause mortality.

**Research question:**

Investigate association between optimism and disease-related characteristics in chronic obstructive pulmonary disease (COPD).

**Study design and methods:**

Current and former smokers with/without COPD and Preserved Ratio Impaired Spirometry (PRISm) from the 10-year follow-up visit for the Genetic Epidemiology of COPD (COPDGene) study were included. Optimism was assessed at the 10-year visit using the Life Orientation Test-Revised. Models of optimism as a predictor of lung function, COPD-associated phenotypes including exacerbations, and functional assessments, were adjusted for demographic confounders, smoking status, and comorbidities.

**Results:**

Among 1967 subjects, higher optimism was significantly associated with older age, non-Hispanic white race, marital status, quitting smoking status, absence of COPD, and absence of depression. In multivariable analysis, higher optimism was independently associated with fewer prior exacerbations of COPD (coef = − 0.037, P < 0.001). Higher optimism was also related to better MMRC scores (coef = − 0.041, P < 0.001), CAT scores (coef = − 0.391, P < 0.001), SGRQ scores (coef = − 0.958, P < 0.001), BODE index (coef = − 0.059, P < 0.001), and longer 6-min walk distance (coef = 10.227, P < 0.001). After stratification by severity of COPD, these associations with optimism were still significant in all groups. No significant association was observed for cross-sectional FEV_1_ (%) or FVC (%) with optimism score.

**Interpretation:**

Fewer exacerbations and less severe respiratory symptoms and higher functional capacity were associated with higher optimism, which may impact health outcomes in current and former smokers with and without COPD. Optimism is a modifiable trait and these results may further support a role for CBT to improve outcomes in COPD.

**Supplementary Information:**

The online version contains supplementary material available at 10.1186/s12931-021-01922-6.

## Introduction

Optimism is a personality trait characterized by the positive expectation that good things will happen in the future [1]. Empirical studies have described that individuals with higher optimism are more likely to be successful [2–6]. Optimism also contributes to health-promoting behaviors, such as smoking cessation, exercise, or healthy diet [2]. Inverse correlation between optimism and depressive symptoms has been observed [7, 8]. Moreover, positive associations between optimism and favorable physical health outcomes, especially related to cardiovascular events and all-cause mortality, were reported in a recent meta-analysis [9]. In the Nurses’ Health Study, all-cause and cause-specific mortality rates were followed prospectively after measuring optimism scores, and women with higher optimism had lower all-cause and cause-specific mortality related to respiratory disease, cancer, heart disease, stroke, and infection [10].

The associations between optimism and pulmonary function were examined prospectively during an average of 8 years of follow-up in the Veterans Administration Normative Aging Study; men with higher optimism had higher levels of lung function and slower rate of lung function decline [11]. Patients with Chronic Obstructive Pulmonary Disease (COPD) often have comorbidities which significantly impact disease course including frequent hospitalization and premature death [12]. Among the comorbidities, depression and anxiety contribute to the burden of COPD and deleterious effects on social functioning, quality of life, and healthcare utilization [13–15]. Furthermore, symptoms of depression and anxiety often overlap with those of COPD, making these comorbid conditions difficult to identify [16]. Though optimism is associated with depression and anxiety [17], the positive health effects of optimism are independent of these psychosocial factors [9–11].

Optimism is partially heritable [18], but also influenced by social structural factors [19]. Several randomized trials have suggested that cognitive behavioral therapy can modify optimism and improve quality of life [20–25]. Nevertheless, the assessment of optimism in clinical practice is uncommon, but represents a potential clinical intervention point for cognitive behavioral therapy to improve outcomes in chronic respiratory diseases. Furthermore, the influence of optimism on lung diseases has not been researched extensively. The aim of our study was to determine the association of higher optimism with less respiratory symptoms and better functional capacity in current and former smokers with and without COPD.

## Materials and methods

### Study design and population

Participants from the 10-year follow-up visit for the Genetic Epidemiology of COPD (COPDGene) cohort were included in the current analysis (N = 1967, data freeze March 20th, 2020, pre-pandemic data). Study design of the COPDGene cohort has been described previously [26]. Briefly, COPDGene is a multicenter observational cohort of non-Hispanic white or African-American current and former smokers in the US, aged between 45 and 80 years at enrollment, with at least 10 pack-years of cigarette smoking. All participants provided written informed consent, and approval was obtained from all 21 local institutional review boards. COPD was diagnosed and classified by the Global Initiative for Chronic Obstructive Lung Disease (GOLD) guidelines [27].

### Measurements

Questionnaires were used to assess sociodemographic information, respiratory symptoms, and medical history at baseline and follow-up visits. Sociodemographic information included age, sex, race, marital status, and smoking history. Respiratory symptoms and health status were assessed using the modified Medical Research Council (mMRC) questionnaire on breathlessness [28, 29], COPD Assessment Test (CAT) [30], and Saint George’s Respiratory Questionnaire (SGRQ) for quality of life (written permission for the use of this tool by COPDGene obtained in 2010) [31]. Higher scores on the SGRQ denote worse quality of life (instrument has not been modified). Exacerbation risk was determined by self-reported history in the previous 12 months and severe exacerbation was defined as requiring a visit to the emergency room or hospitalization. Spirometry and 6-min walk distance tests were performed at each visit according to standard techniques [32, 33], and the BODE index was calculated using these measurements [34]. GOLD grade for airflow limitation was classified using post-bronchodilator spirometry among subjects with forced expiratory volume in 1 s (FEV_1_)/forced vital capacity (FVC) < 0.7: GOLD 1, FEV_1_ ≥ 80% predicted; GOLD 2, 50% ≤ FEV_1_ < 80% predicted; GOLD 3, 30% ≤ FEV_1_ < 50% predicted; and GOLD 4, FEV_1_ < 30% predicted [27]. Subjects with Preserved Ratio Impaired Spirometry (PRISm) were defined as having FEV_1_/FVC ≥ 0.7 and FEV_1_ < 80% predicted. Smokers with normal spirometry were defined by FEV_1_/FVC > 0.7 and FEV_1_ > 80% predicted. Optimism was measured at the 10-year follow-up visit using the Life Orientation Test-Revised (LOT-R) [35] which is composed of 6 validated items: three items to assess optimism and three items to assess pessimism. Each item is on a Likert scale (score options 0–4), and the total score is calculated by adding the optimism question scores and inverted pessimism question scores, as published previously [35]. Total score ranges from 0 to 24, and higher scores indicate higher optimism. To assess the possibility of discontinuous or threshold effects, quartiles of optimism based on score distributions were created. Depression and anxiety were assessed as self-reported history of doctor-diagnosed depression and anxiety. The Hospital Anxiety and Depression Scale (HADS), which can measure both anxiety and depression in a general medical population, was also obtained [36]. HADS is composed of 7 items for depression, with each item ranging from 0 to 3, with total scores ranging from 0 to 21. We used the cut-off value of HADS score of 8 points to categorize depression at the time of study visit evaluation. A flow chart summarizing the covariates and predictors at each study phase is provided (Additional file 1: Fig. S1).

### Statistical analysis

Subject characteristics are presented as means (± SD) for continuous variables and as relative frequencies for categorical variables. Means were compared using a t-test or analysis of variance (ANOVA), and categorical variables were compared using a chi-squared or Fisher’s exact test. Univariable analysis for association between variables and optimism was performed stratified according to GOLD stage. For the occurrence of severe exacerbations and number of exacerbations, logistic regression models and Poisson regression models were used, respectively. For the MMRC, CAT, SGRQ, and 6-min walk distance, linear regression was used for each model. To evaluate multivariable associations with optimism, two models were evaluated. Model 1 adjusted for demographics (DM) including age, sex, race, and marital status and added health behavior factors (HB): body mass index (BMI) and current smoking status as potential confounders (DM + HB). Model 2 further adjusted for health conditions (HC) adding presence of depression, hypertension, diabetes mellitus, and FEV_1_ (% predicted) to model 1 (DM + HB + HC). Optimism score was examined both as a continuous and as a categorical variable (quartiles). Regression models for the association of optimism with lung function including FEV_1_ (% predicted) or FVC (% predicted) were adjusted for age, sex, race, marital status, BMI, current smoking status, presence of depression, hypertension, and diabetes mellitus in model 2. To evaluate whether functional status would predict optimism at visit 3, values of MMRC, CAT, SGRQ score, or 6-min walk distance at visit 2(5 years) or visit 1(baseline) were adjusted for age, sex, race, marital status, BMI, current smoking status, history of depression, presence of hypertension, diabetes mellitus, and FEV_1_ (% predicted) at visit 2 or visit 1, respectively. BODE index models excluded variables for BMI and FEV_1_ (% predicted) in model 2. All the statistical analysis was performed using R (version 3.6.0).

## Results

**Fig. 1:**
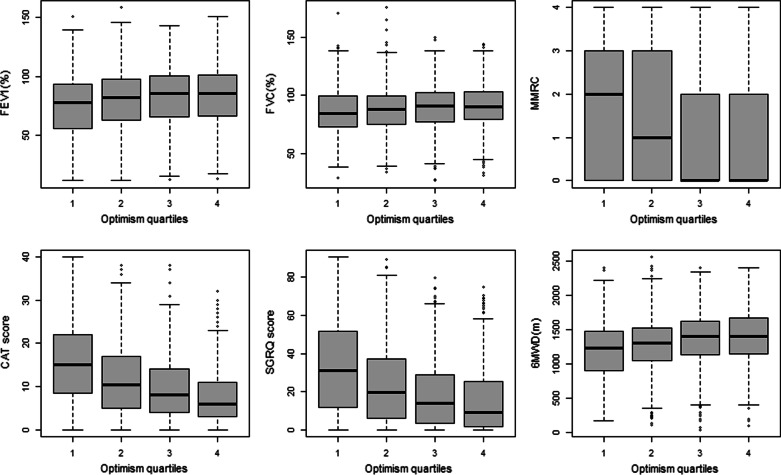
Univariable comparison of lung function and functional capacities among optimism quartiles

**Fig. 2:**
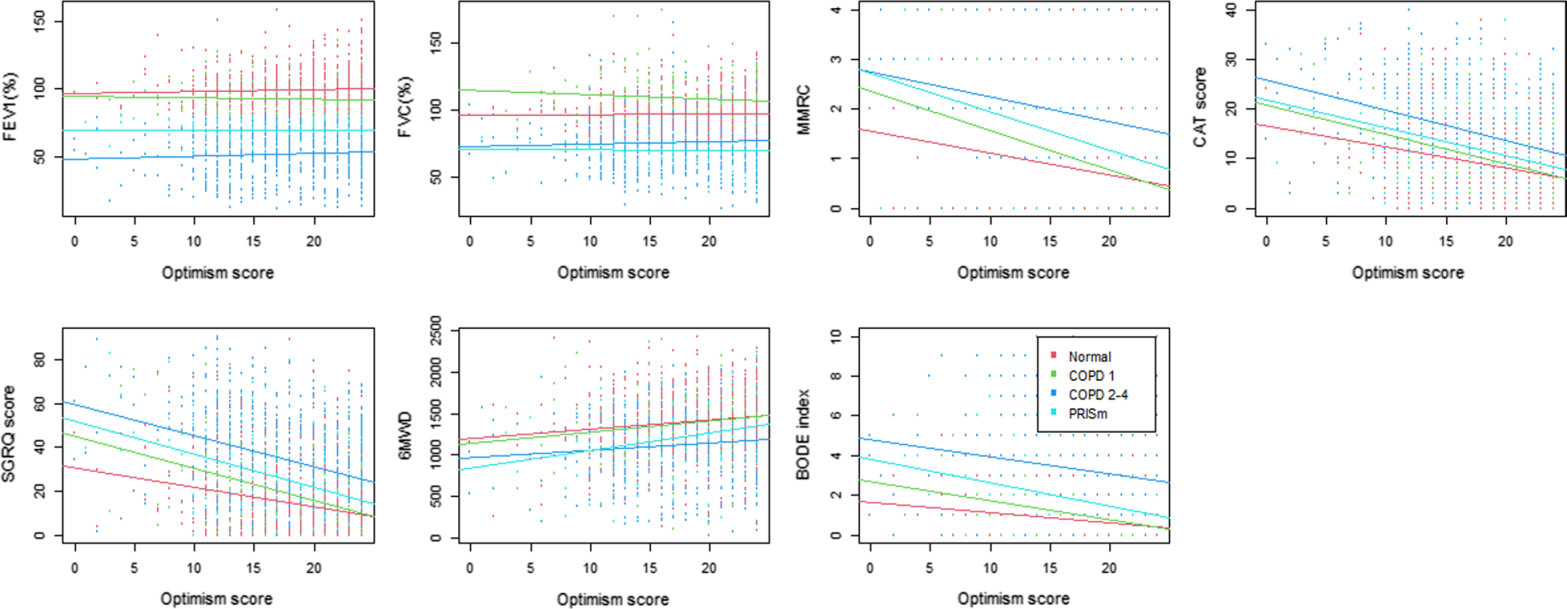
Correlation of lung function and functional capacities with optimistic score stratified by severity of airflow limitation

A total of 1967 subjects returned for the ten-year follow-up of COPDGene as of March 2020 (pre-pandemic shutdown), consisting of 812 without spirometric airflow limitation, 241 GOLD 1, 384 GOLD 2, 193 GOLD 3, 101 GOLD 4, and 236 PRISm. Demographic and clinical characteristics of these participants are summarized in Table 1. Mean optimism scores according to spirometry stage were 18.9, 18.3, 17.8, and 17.6 for normal spirometry, GOLD 1, GOLD 2–4, and PRISm, respectively. Subjects with COPD GOLD 2–4 presented with lower optimism than those with normal spirometry (coef = − 0.915, P < 0.001). Higher optimism score was associated with advanced age, non-Hispanic white race, being married, former smoking status, and absence of depression or anxiety in all stratified groups in univariable analysis. However, there was no significant association with pulmonary function including FEV_1_ (% predicted) and FVC (% predicted) in all stratified groups (Table 2). Significant associations between higher optimism score with fewer respiratory symptoms and better MMRC, CAT, SGRQ score, 6-min walk distance, and BODE index were present in univariable analysis (Table 2 and Figs. 1, 2). Fewer and less severe prior COPD exacerbations were observed in the 3rd and 4th highest optimism quartile groups in univariable analysis (Table 3). For the prior severe exacerbation events, in model 1 adjusted for demographics and health behavior (DM + HB), these associations remained significant, but in model 2, after further adjustment for other health conditions (DM + HB + HC), the association was attenuated but the effect was in the same direction. For the number of prior exacerbations, model 1 (DM + HB) was significant in the 2nd, 3rd, and 4th optimism quartile groups compared to the lowest quartile. In model 2 (DM + HB + HC), the associations were substantially attenuated but remained significant in the 3rd and 4th quartile: high optimism group. Higher frequency of prior history of exacerbations was significantly associated with lower optimism score as a continuous value (Table 3). For the functional outcomes, higher optimism was independently associated with less severe MMRC (coef = − 0.041, P < 0.001), CAT (coef = − 0.391, P < 0.001), and total SGRQ score (coef = − 0.958, P < 0.001) including all scores of each domain (activity domain, coef = − 1.125, P < 0.001; impact domain, coef = − 0.908, P < 0.001; symptom domain, coef = − 0.759, P < 0.001), BODE index (coef = − 0.059, P < 0.001), and higher 6-min walk distance (coef = 10.227, P < 0.001) in the fully adjusted model.

**Table 1:** Characteristics of study participants

	Normal spirometry (N = 812)	GOLD 1 (N = 241)	GOLD 2–4 (N = 678)	PRISm (N = 236)	P-value (normal vs. GOLD 2–4)	P-value (normal vs PRISm)
Age	67.32 ± 8.07	71.77 ± 9.01	71.27 ± 8.10	65.27 ± 7.33	< 0.001	< 0.001
Female sex (n, %)	438 (53.9%)	112 (46.5%)	319 (47.1%)	126 (53.4%)	0.009	0.940
White race (n, %)	550 (67.7%)	184 (76.3%)	526 (77.6%)	120 (50.8%)	< 0.001	< 0.001
BMI (mean ± SD)	28.78 ± 5.88	26.86 ± 5.31	27.83 ± 6.37	32.05 ± 7.02	0.003	< 0.001
Marital status					0.248	0.005
Married	349 (43.0%)	107 (44.4%)	276 (40.7%)	64 (27.1%)		
Divorced	190 (23.4%)	51 (21.2%)	175 (25.8%)	61 (25.8%)		
Widowed	70 (8.6%)	38 (15.8%)	96 (14.2%)	33 (14.0%)		
Separated	23 (2.8%)	4 (1.7%)	13 (1.9%)	9 (3.8%)		
Never	156 (19.2%)	34 (14.1%)	108 (15.9%)	67 (28.4%)		
Unmarried couple	22 (2.7%)	7 (2.9%)	10 (1.5%)	2 (0.8%)		
Current smoker	322 (39.7%)	92 (38.2%)	246 (36.3%)	118 (50.0%)	0.119	0.008
Comorbidities						
Hypertension	423 (52.1%)	137 (56.8%)	428 (63.1%)	158 (66.9%)	< 0.001	< 0.001
Diabetes	160 (19.7%)	43 (17.8%)	130 (19.2%)	83 (35.2%)	0.848	< 0.001
Depression	213 (26.2%)	55 (22.8%)	165 (24.3%)	76 (32.2%)	0.437	0.085
Anxiety	159 (19.6%)	43 (17.8%)	135 (19.9%)	58 (24.6%)	0.925	0.115
HADS-depression	2.68 ± 2.91	3.16 ± 3.22	3.85 ± 3.15	3.57 ± 3.27	< 0.001	< 0.001
HADS-anxiety	3.57 ± 3.43	3.83 ± 3.49	4.03 ± 3.64	4.55 ± 3.80	0.012	< 0.001
Lung function						
FEV_1_ (%)	99.45 ± 13.53	92.59 ± 10.47	51.75 ± 17.24	69.42 ± 8.52	< 0.001	< 0.001
FVC (%)	96.90 ± 13.22	108.78 ± 14.87	75.89 ± 17.87	69.94 ± 9.57	< 0.001	< 0.001
FEV_1_/FVC	0.78 ± 0.05	0.64 ± 0.05	0.51 ± 0.12	0.76 ± 0.05	< 0.001	< 0.001

**Table 2:** Univariable analysis for optimism score

Optimism score	Total (N = 1967)		Normal spirometry (N = 812)		GOLD 1 (N = 241)		GOLD 2–4 (N = 678)		PRISm (N = 236)	
	Coef.	P-value	Coef.	P-value	Coef.	P-value	Coef.	P-value	Coef.	P-value
Age	0.109	< 0.001	0.107	< 0.001	0.104	0.003	0.146	< 0.001	0.154	< 0.001
Female Sex	0.296	0.173	0.632	0.049	− 0.186	0.769	0.123	0.750	− 0.222	0.730
White race	1.951	< 0.001	2.112	< 0.001	2.916	< 0.001^5^	1.653	< 0.001	1.82	0.004
BMI	− 0.006	0.722	0.061	0.026	-0.100	0.093	− 0.028	0.348	− 0.038	0.399
Marital status^a^	0.440	< 0.001	1.264	< 0.001	2.127	< 0.001	1.200	0.002	1.546	0.017
Current smoking	− 2.004	< 0.001	− 2.212	< 0.001	− 0.994	0.131	− 2.076	< 0.001	− 2.156	< 0.001
Comorbidities										
Depression	− 2.670	< 0.001	− 1.839	< 0.001	− 2.857	< 0.001	− 3.538	< 0.001	− 2.927	< 0.001
Anxiety	− 2.529	< 0.001	− 1.782	< 0.001	− 2.725	< 0.001	− 3.160	< 0.001	− 2.805	< 0.001
HADS-depression	− 0.804	< 0.001	− 0.724	< 0.001	− 0.871	< 0.001	− 0.887	< 0.001	− 0.697	< 0.001
HADS-anxiety	− 0.675	< 0.001	− 0.575	< 0.001	− 0.806	< 0.001	− 0.750	< 0.001	− 0.593	< 0.001
Hypertension	− 0.586	0.008	− 0.732	0.022	− 1.040	0.103	0.023	0.953	− 0.288	0.672
Diabetes	− 1.105	< 0.001	− 0.799	0.047	− 1.961	0.017	− 1.090	0.025	− 0.944	0.158
Lung function										
FEV_1_ (% predict)	0.020	< 0.001	0.016	0.182	− 0.025	0.408	0.020	0.075	0.008	0.826
FVC (% predict)	0.016	0.004	0.003	0.828	− 0.037	0.084	0.013	0.241	− 0.015	0.647
FEV_1_/FVC	2.159	0.003	− 0.227	0.943	0.352	0.956	0.829	0.595	− 1.412	0.837
Functional status										
MMRC	− 0.768	< 0.001	− 0.727	< 0.001	− 1.249	< 0.001	− 0.644	< 0.001	− 1.030	< 0.001
CAT	− 0.201	< 0.001	− 0.183	< 0.001	− 0.261	< 0.001	− 0.214	< 0.001	− 0.195	< 0.001
SGRQ	− 0.078	< 0.001	− 0.075	< 0.001	− 0.118	< 0.001	− 0.079	< 0.001	− 0.093	< 0.001
6MWD	1.98E−03	< 0.001	0.002	< 0.001	0.002	0.015	0.001	0.007	0.003	< 0.001
BODE	− 0.493	< 0.001	− 0.821	< 0.001	− 1.384	< 0.001	− 0.321	< 0.001	− 1.011	< 0.001

^a^Marital status: divorced, separated, never, unmarried couple status compared to married or widowed status; Optimism scores are examined as continuous variable

**Table 3:** Association of optimism and past exacerbation history of COPD in all subjects

Severe exacerbation	Q2 vs. Q1 for optimism score		Q3 vs. Q1 for optimism score		Q4 vs. Q1 for optimism score		Optimism as a continuous variable	
	OR	95% CI	OR	95% CI	OR	95% CI	OR	95% CI
Unadjusted	0.695	0.465–1.039	0.457	0.286–0.729	0.530	0.341–0.824	0.943	0.914–0.972
DM + HB	0.686	0.454–1.036	0.462	0.289–0.748	0.525	0.330–0.835	0.941	0.911–0.971
DM + HB + HC	0.869	0.561–1.344	0.685	0.411–1.142	0.792	0.481–1.302	0.970	0.936–1.005

Exacerbation frequency	Coef.	P-value	Coef.	P-value	Coef.	P-value	Coef.	P-value
Unadjusted	− 0.325	0.005	− 0.665	5.55 × 10^–7^	− 0.863	6.23 × 10^–10^	− 0.061	2.85 × 10^–12^
DM + HB	− 0.354	0.002	− 0.756	2.32 × 10^–8^	− 0.992	5.46 × 10^–12^	− 0.068	1.48 × 10^–14^
DM + HB + HC	− 0.088	0.457	− 0.334	0.017	− 0.542	2.69 × 10^–4^	− 0.037	1.39 × 10^–4^

Q4: most optimistic

Demographic: Age, sex, race, marital status

Health Behavior: BMI, smoking status

Health condition: Depression, hypertension, diabetes, FEV_1_ (% predicted)

### Associations between optimism and COPD

We stratified the study population according to severity of airflow limitation as normal spirometry, GOLD 2–4, and PRISm groups using GOLD grade, and evaluated the association of optimism with MMRC score, CAT score, all domains of SGRQ score, 6-min walk distance, and BODE index—all were statically significant in the stratified groups (Table 4). Detailed results of each model are summarized (Additional file 1: Table S1). Effects sizes were slightly attenuated but the directions of effect were similar regardless of adjustment for doctor-diagnosed history of depression or adjustment using cut-off value of HADS (Additional file 1: Table S2). To address potential concerns regarding residual confounding between optimism and depression, we repeated the same analysis excluding respondents with previous history of depression. Association between optimism and MMRC, CAT score, all domains of SGRQ score, 6-min walk distance and BODE index were still significant in the presence of COPD (GOLD 1–4). In subjects with normal spirometry, association with CAT score and SGRQ score remained robust after the most stringent removal of subjects with previous history of depression (Additional file 1: Tables S3 and S4). No significant associations between optimism score and lung function were observed for the multivariable analysis (Table 4 and Additional file 1: Tables S1–S3).

**Table 4:** Multivariate association of optimism with lung function and functional outcomes (Model 2)

	Normal spirometry		GOLD 1		GOLD 2–4		PRISm	
	Coef.	P-value	Coef.	P-value	Coef.	P-value	Coef.	P-value
FEV_1_ (% predicted)	0.055	0.611	− 0.256	0.700	0.319	0.025	− 0.034	0.785
FVC (% predicted)	0.055	0.611	− 0.460	0.023	0.175	0.241	− 0.076	0.586
MMRC score	− 0.031	< 0.001	− 0.051	< 0.001	− 0.038	< 0.001	− 0.048	0.008
CAT score	− 0.278	< 0.001	− 0.390	< 0.001	− 0.430	< 0.001	− 0.429	< 0.001
SGRQ Total score	− 0.669	< 0.001	− 1.038	< 0.001	− 1.014	< 0.001	− 1.103	< 0.001
SGRQ Activity	− 0.951	< 0.001	− 1.205	< 0.001	− 0.966	< 0.001	− 1.433	< 0.001
SGRQ Impact	− 0.511	< 0.001	− 0.959	< 0.001	− 1.141	< 0.001	− 0.940	< 0.001
SGRQ Symptoms	− 0.639	< 0.001	− 0.954	< 0.001	− 0.595	< 0.001	− 0.995	0.003
6-min walk distance	9.585	< 0.001	8.639	0.092	7.958	0.004	17.509	< 0.001
BODE index	− 0.036	< 0.001	− 0.070	< 0.001	− 0.053	< 0.001	− 0.079	< 0.001

Adjusted by age, sex, race, marital status, BMI, current smoking status, presence of depression, hypertension, diabetes, and FEV_1_ (% predicted)

### Associations between optimism and PRISm

Baseline characteristics of PRISm subjects are summarized in Table 1. The associations between higher optimism score and functional capacities were in the same direction as for COPD subjects. In multivariable analysis, all these outcomes remained significant but suggested stronger association in PRISm subjects, especially for 6-min walk distance. Results of the sensitivity analysis excluding subjects with history of doctor-diagnosed depression are summarized (Additional file 1: Table S3). After adjustment for cut-off value of HADS instead of previous history of depression diagnosis, the results were similar (Additional file 1: Tables S2 and S4).

### Predictive variables for future optimism

Values of MMRC, CAT, SGRQ score, 6-min walk distance and BODE index at visit 1 and 2 were significantly associated with optimism scores at visit 3 in multivariable analysis, and changes of MMRC, CAT, SGRQ score, and BODE index 5 and 10 years previously were also significantly associated with visit 3 optimism in smokers with and without COPD (Additional file 1: Table S5). The association between visit 2 functional variables and visit 3 optimism scores were examined at each group stratified by GOLD grade. CAT, SGRQ score and 6-min walk distance were significant predictors for optimism score at visit 3 in all groups. For GOLD 2–4 groups, decline of CAT, or SGRQ score between visit 2 and visit 3 were associated with lower optimism score at visit 3. Association of optimism score with visit 1 functional capacities and their changes are also summarized in Additional file 1: Table S5. In the PRISm population, MMRC, CAT, SGRQ score, 6-min walk distance, and BODE index at visit 2 were all significant predictor for optimism score at visit 3, but no significant association was observed for changes during follow-up.

## Discussion

We found significant associations between optimism and respiratory symptoms and functional status including MMRC, CAT score, SGRQ score, 6-min walk distance, and BODE index in the presence or absence of COPD in current and former smokers. Higher optimism was related to fewer respiratory symptoms, less frequent report of prior exacerbations of COPD, higher exercise capacity, and better quality of life. Optimism is a modifiable factor, with strong associations with health status and mortality. It is well known that respiratory symptoms or quality of life in COPD correlate poorly with lung function [37]. Cognitive behavioral therapy (CBT) for patients with COPD has been suggested to improve their anxiety, depression [38], breathlessness [39], and quality of life [40]. Traditional CBT for mood disorders focuses on problem solving. However, positive CBT for optimism focuses on feeling positive emotion, being engaged at home and work, and experiencing positive relationships and accomplishments [41]; investigating optimism may provide an innovative intervention opportunity in conjunction with pulmonary rehabilitation to improve quality of life and outcomes in COPD [42].

Severity of COPD is classified based on occurrence of exacerbations in the past 12 months and functional status including symptoms and quality of life, in addition to lung function [27]. Our study revealed that a positive outlook may impact COPD functional outcomes; addressing psychosocial aspects of COPD constitutes an important and undertreated aspect of COPD management. Psychological factors may exert their effects by both the indirect behavioral mechanisms and the direct physiologic mechanisms [43]. Physical activity, healthier diet, and smoking cessation are favorable health behaviors associated with optimism [44]. More active problem-solving tendencies of optimistic people may be advantageous to mitigate risk of exacerbation, cope with and relieve stress, and promote better overall health quality [45]. In addition, optimism may impact medication compliance for stable COPD. Furthermore, optimism could potentially influence COPD pathobiology directly through modulation of immune function [46, 47] by various mediators [48, 49]. Since COPD is a chronic inflammatory lung disease, those pathophysiologic mechanisms could directly affect functional capacities and pulmonary outcomes. Heritability of optimism has been estimated as 25% [18]; in other words, although having genetic features optimism can be learned and influenced by underlying health conditions. However, optimism also demonstrates stability across a long span of time without intervention [50], and has been associated with positive health effects in many prospective studies.

There are limitations to our current study. It is not clear whether the association of optimism with functional outcomes is a cause or effect of prior respiratory status. We cannot completely rule out the possibility that a confounding factor links optimism and functional outcomes, although we adjusted for several potential confounders, especially depression, in multiple ways and these results remained robust after stringent adjustment for depression. Optimism scores were only available at the 10-year follow-up visit, so we could not assess impact of optimism score prospectively. A published prospective study described men with higher optimism associated with higher lung function and slower rate of lung function decline over course of follow-up in the Normative Aging Study [9]. However, we did not observe a longitudinal relationship between prior lung function decline and optimism in our study population. Given that subjects in COPDGene were ascertained to have high smoking histories and more severe COPD subjects, our results may not be generalizable to the general population. In addition, our study included non-Hispanic white and African Americans, and therefore might not be generalized to Hispanic or Asian individuals with a history of smoking.

In summary, higher dispositional optimism is associated with fewer COPD exacerbations and better functional status in smokers with and without COPD. Future research should be focused upon replicating our findings, but these results may further support a role of cognitive behavioral therapy to improve outcomes for COPD.

## Supplementary Information

**Additional file 1: Table S1.** Multivariate association of optimism with lung function and functional outcomes with 2 tier model. **Table S2.** HADS adjustment instead of previous history of depression diagnosis. **Table S3.** Sensitivity analysis excluding subjects with history of depression. **Table S4.** Sensitivity analysis excluding collection of subjects with previous history of depression and present depression by HADS. **Table S5.** Association of disease-related characteristics at previous visits as predictor of optimism score in next visit. **Fig. S1.** Flow chart of enrollment of COPDGene study population.

## Data Availability

COPDGene data are available through dbGAP (dbGaP Study Accession: phs000179.v6.p2).

## Abbreviations

BMI: Body mass index
CAT: COPD Assessment Test
CBT: Cognitive behavioral therapy
COPD: Chronic Obstructive Pulmonary Disease
COPDGene: Genetic Epidemiology of COPD
FEV_1_: Forced expiratory volume in 1s
FVC: Forced vital capacity
GOLD: Global Initiative for Chronic Obstructive Lung Disease
HADS: Hospital Anxiety and Depression Scale
LOT-R: The Life Orientation Test-Revised
mMRC: Modified Medical Research Council
PRISm: Preserved Ratio Impaired Spirometry
SGRQ: Saint George’s Respiratory Questionnaire
