# Supplementary material for: Optimism is associated with respiratory symptoms and functional status in chronic obstructive pulmonary disease
Source: Respir Res. 2022 Jan 29;23:19. doi: 10.1186/s12931-021-01922-6 (PMC8800351; doi:10.1186/s12931-021-01922-6)
Supplement: Supplementary file 1 — Additional file 1: Table S1. Multivariate association of optimism with lung function and functional outcomes with 2 tier model. Table S2. HADS adjustment instead of previous history of depression diagnosis. Table S3. Sensitivity analysis excluding subjects with history of depression. Table S4. Sensitivity analysis excluding collection of subjects with previous history of depression and present depression by HADS. Table S5. Association of disease-related characteristics at previous visits as predictor of optimism score in next visit. Fig. S1. Flow chart of enrollment of COPDGene study population. [file 12931_2021_1922_MOESM1_ESM.docx]

Table S1. Multivariate association of optimism with lung function and functional outcomes with 2 tier model

|  | **COPD** | | | | **PRISm** | | | |
| --- | --- | --- | --- | --- | --- | --- | --- | --- |
|  | **DM+HB** | | **DM+HB+HC** | | **DM+HB** | | **DM+HB+HC** | |
|  | Coef | P-value | Coef | P-value | Coef | P-value | Coef | P-value |
| **FEV_1_ (% predicted)** | 0.059 | 0.579 | 0.055 | 0.611 | -0.222 | 0.106 | -0.256 | 0.700 |
| **FVC (% predicted)** | 0.059 | 0.579 | 0.055 | 0.611 | -0.390 | 0.047 | -0.460 | 0.023 |
| **MMRC score** | -0.043 | <0.001 | -0.031 | <0.001 | -0.055 | <0.001 | -0.051 | <0.001 |
| **CAT score** | -0.320 | <0.001 | -0.278 | <0.001 | -0446 | <0.001 | -0.390 | <0.001 |
| **SGRQ Total score** | -0.779 | <0.001 | -0.669 | <0.001 | -1.168 | <0.001 | -1.038 | <0.001 |
| **SGRQ Activity** | -1.121 | <0.001 | -0.951 | <0.001 | -1.427 | <0.001 | -1.205 | <0.001 |
| **SGRQ Impact** | -0.590 | <0.001 | -0.511 | <0.001 | -1.039 | <0.001 | -0.959 | <0.001 |
| **SGRQ Symptoms** | -0.738 | <0.001 | -0.639 | <0.001 | -1.076 | <0.001 | -0.954 | <0.001 |
| **6-minute walk distance** | 11.00 | <0.001 | 9.585 | <0.001 | 10.044 | 0.043 | 8.639 | 0.092 |
| **BODE index** | -0.045 | <0.001 | -0.036 | <0.001 | -0.076 | <0.001 | -0.070 | <0.001 |

|  | COPD | | | | PRISm | | | |
| --- | --- | --- | --- | --- | --- | --- | --- | --- |
|  | DM+HB | | DM+HB+HC | | DM+HB | | DM+HB+HC | |
|  | Coef | P-value | Coef | P-value | Coef | P-value | Coef | P-value |
| **FEV_1_ (% predicted)** | 0.298 | 0.029 | 0.319 | 0.025 | -0.006 | 0.958 | -0.034 | 0.785 |
| **FVC (% predicted)** | 0.144 | 0.315 | 0.175 | 0.241 | -0.049 | 0.710 | -0.076 | 0.586 |
| **MMRC score** | -0.054 | <0.001 | -0.038 | <0.001 | -0.64 | <0.001 | -0.048 | 0.008 |
| **CAT score** | -0.548 | <0.001 | -0.430 | <0.001 | -0.475 | <0.001 | -0429 | <0.001 |
| **SGRQ Total score** | -1.356 | <0.001 | -1.014 | <0.001 | -1.303 | <0.001 | -1.103 | <0.001 |
| **SGRQ Activity** | -1.401 | <0.001 | -0.966 | <0.001 | -1.767 | <0.001 | -1.433 | <0.001 |
| **SGRQ Impact** | -1.435 | <0.001 | -1.141 | <0.001 | -1.100 | <0.001 | -0.94 | <0.001 |
| **SGRQ Symptoms** | -0.922 | <0.001 | -0.595 | <0.001 | -1.047 | 0.001 | -0.995 | 0.003 |
| **6-minute walk distance** | 11.383 | <0.001 | 7.958 | 0.004 | 18.520 | <0.001 | 17.509 | <0.001 |
| **BODE index** | -0.093 | <0.001 | -0.053 | <0.001 | -0.096 | <0.001 | -0.079 | <0.001 |

Demographic model (DM): Age, sex, race, marital status

Health Behavior model (HB): BMI, smoking status

Health condition (HC): Depression, hypertension, diabetes, FEV_1_ (% predicted)

Table S2. HADS adjustment instead of previous history of depression diagnosis

|  | **Normal Spirometry (N=413)** | | **GOLD 1**  **(N=113)** | | **GOLD 2-4**  **(N = 360)** | | **PRISm**  **(N=114)** | |
| --- | --- | --- | --- | --- | --- | --- | --- | --- |
|  | Coef | P-value | Coef | P-value | Coef | P-value | Coef | P-value |
| **FEV_1_, %predicted†** | 0.098 | 0.381 | -0.193 | 0.194 | 0.205 | 0.174 | -0.009 | 0.941 |
| **FVC, %predicted†** | 0.089 | 0.414 | -0.375 | 0.080 | 0.111 | 0.483 | -0.081 | 0.570 |
| **MMRC score‡** | -0.028 | 0.002 | -0.048 | 0.003 | -0.021 | 0.042 | -0.038 | 0.037 |
| **CAT score‡** | -0.253 | <0.001 | -0.295 | 0.001 | -0.316 | <0.001 | -0.377 | 0.001 |
| **SGRQ score‡** | -0.506 | <0.001 | -0.716 | <0.001 | -0.653 | <0.001 | -0.950 | <0.001 |
| **SGRQ Activity** | -0.732 | <0.001 | -0.800 | 0.020 | -0.596 | 0.002 | -1.333 | <0.001 |
| **SGRQ Impact** | -0.348 | <0.001 | -0.676 | <0.001 | -0.756 | <0.001 | -0.765 | 0.001 |
| **SGRQ Symptom** | -0.595 | <0.001 | -0.682 | 0.022 | -0.367 | 0.041 | -0.801 | 0.017 |
| **6MWD‡** | 7.179 | 0.016 | 2.731 | 0.601 | 4.616 | 0.114 | 16.298 | 0.002 |
| **BODE index‡** | -0.032 | <0.001 | -0.053 | 0.001 | -0.029 | 0.034 | -0.068 | <0.001 |

†Adjusted by age, sex, race, marital status, BMI, depression, current smoking status, presence of COPD, HTN, and diabetes mellitus

‡Adjusted by age, sex, race, marital status, BMI, depression, current smoking status, presence of COPD, HTN, diabetes mellitus, and FEV_1_ (%) predicted

Table S3. Sensitivity analysis excluding subjects with history of depression

|  | **Normal Spirometry (N=599)** | | **GOLD 1**  **(N=186)** | | **GOLD 2-4**  **(N = 513)** | | **PRISm**  **(N=160)** | |
| --- | --- | --- | --- | --- | --- | --- | --- | --- |
|  | Coef | P-value | Coef | P-value | Coef | P-value | Coef | P-value |
| **FEV_1_, %predicted†** | 0.104 | 0.444 | -0.098 | 0.573 | 0.231 | 0.211 | -0.084 | 0.621 |
| **FVC, %predicted†** | 0.101 | 0.440 | -0.463 | 0.069 | 0.136 | 0.477 | -0.180 | 0.329 |
| **MMRC score‡** | -0.013 | 0.194 | -0.050 | 0.007 | -0.042 | 0.001 | -0.014 | 0.549 |
| **CAT score‡** | -0.246 | <0.001 | -0.422 | <0.001 | -0.423 | <0.001 | -0.198 | 0.173 |
| **SGRQ score‡** | -0.563 | <0.001 | -1.160 | <0.001 | -0.908 | <0.001 | -0.533 | 0.097 |
| **SGRQ Activity** | -0.848 | <0.001 | -1.489 | <0.001 | -1.008 | <0.001 | -0.904 | 0.066 |
| **SGRQ Impact** | -0.395 | <0.001 | -0.999 | <0.001 | -0.928 | <0.001 | -0.330 | 0.238 |
| **SGRQ Symptom** | -0.581 | 0.003 | -1.016 | 0.003 | -0.574 | 0.009 | -0.495 | 0.254 |
| **6MWD‡** | 6.482 | 0.072 | 17.907 | 0.003 | 10.320 | 0.002 | 12.066 | 0.081 |
| **BODE index‡** | -0.013 | 0.195 | -0.074 | <0.001 | -0.058 | <0.001 | -0.055 | 0.024 |

Adjusted for age, sex, race, marital status, BMI, current smoking history, hypertension, diabetes, FEV_1_ (% predicted)

Table S4. Sensitivity analysis excluding collection of subjects with previous history of depression and present depression by HADS

|  | **Normal Spirometry (N=291)** | | **GOLD 1**  **(N=84)** | | **GOLD 2-4**  **(N = 256)** | | **PRISm**  **(N=71)** | |
| --- | --- | --- | --- | --- | --- | --- | --- | --- |
|  | Coef | P-value | Coef | P-value | Coef | P-value | Coef | P-value |
| **FEV_1_ (% predicted)** | 0.140 | 0.325 | -0.025 | 0.898 | 0.236 | 0.225 | -0.097 | 0.583 |
| **FVC (% predicted)** | 0.111 | 0.418 | -0.373 | 0.179 | 0.070 | 0.727 | -0.216 | 0.263 |
| **MMRC score** | -0.008 | 0.441 | -0.032 | 0.102 | -0.030 | 0.022 | -0.007 | 0.776 |
| **CAT score** | -0.230 | <0.001 | -0.298 | 0.009 | -0.326 | <0.001 | -0.179 | 0.217 |
| **SGRQ Total score** | -0.477 | <0.001 | -0.620 | 0.009 | -0.708 | <0.001 | -0.450 | 0.116 |
| **SGRQ Activity** | -0.700 | 0.002 | -0.802 | 0.047 | -0.798 | <0.001 | -0.786 | 0.112 |
| **SGRQ Impact** | -0.325 | 0.002 | -0.521 | 0.002 | -0.739 | <0.001 | -0.236 | 0.382 |
| **SGRQ Symptoms** | -0.577 | 0.004 | -0.589 | 0.086 | -0.362 | 0.114 | -0.517 | 0.242 |
| **6-minute walk distance** | 5.075 | 0.182 | 12.825 | 0.042 | 8.313 | 0.015 | 11.580 | 0.106 |
| **BODE index** | -0.012 | 0.264 | -0.041 | 0.024 | -0.043 | 0.008 | -0.052 | 0.037 |

Table S5. Association of disease-related characteristics at previous visits as predictor of optimism score in next visit

|  | **Visit 1 values** | | **Visit 2 values** | | **Changes btw visit 1 and visit 3** | | **Changes btw visit 2 and visit 3** | |
| --- | --- | --- | --- | --- | --- | --- | --- | --- |
|  | Coef | P-value | Coef | P-value | Coef | P-value | Coef | P-value |
| **Total** |  |  |  |  |  |  |  |  |
| **MMRC** | -0.416 | <0.001 | -0.376 | <0.001 | -0.232 | 0.004 | -0.333 | <0.001 |
| **CAT** | − | − | -0.108 | <0.001 | − | − | -0.066 | <0.001 |
| **SGRQ** | -0.044 | <0.001 | -0.046 | <0.001 | -0.029 | <0.001 | -0.044 | <0.001 |
| **6MWD** | 0.002 | <0.001 | 0.002 | <0.001 | 1.86 x10^-04^ | 0.553 | 1.79 x10^-04^ | 0.594 |
| **BODE** | -0.411 | <0.001 | -0.332 | <0.001 | -0.292 | <0.001 | -0.386 | <0.001 |
| **Normal Spirometry** | | | | | | | | |
| **MMRC** | -0.384 | 0.018 | -0.313 | 0.060 | -0.146 | 0.287 | -0.269 | 0.087 |
| **CAT** | − | − | -0.085 | <0.001 | − | − | -0.049 | 0.047 |
| **SGRQ** | -0.040 | <0.001 | -0.046 | <0.001 | -0.017 | 0.115 | -0.023 | 0.069 |
| **6MWD** | 0.001 | 0.019 | 0.001 | 0.006 | 6.43 x10^-04^ | 0.187 | 3.43 x10^-04^ | 0.483 |
| **BODE** | -0.406 | 0.022 | -0.257 | 0.129 | -0.352 | 0.019 | -0.543 | 0.002 |
| **GOLD 2-4** | | | | | | | | |
| **MMRC** | -0.307 | 0.045 | -0.089 | 0.567 | -0.195 | 0.128 | -0.392 | 0.005 |
| **CAT** | − | − | -0.069 | 0.007 | − | − | -0.094 | <0.001 |
| **SGRQ** | -0.037 | <0.001 | -0.030 | 0.005 | -0.027 | 0.008 | -0.006 | <0.001 |
| **6MWD** | 0.002 | <0.001 | 0.001 | 0.028 | -7.37 x10^-04^ | 0.164 | 8.49 x10^-05^ | 0.890 |
| **BODE** | -0.380 | 0.005 | -0.193 | 0.140 | -0.092 | 0.353 | -0.163 | 0.184 |
| **PRISm** | | | | | | | | |
| **MMRC** | -0.534 | 0.030 | -0.672 | 0.013 | -0.170 | 0.433 | -0.258 | 0.269 |
| **CAT** | − | − | -0.135 | 0.001 | − | − | -0.053 | 0.248 |
| **SGRQ** | -0.039 | 0.014 | -0.052 | 0.002 | -0.032 | 0.081 | -0.026 | 0.203 |
| **6MWD** | 0.002 | 0.104 | 0.003 | 0.002 | 0.001 | 0.192 | -3.31 x10^-04^ | 0.742 |
| **BODE** | -0.534 | 0.035 | -0.400 | 0.085 | -0.362 | 0.098 | -0.474 | 0.044 |

Adjusted by age, sex, race, marital status, BMI, depression, current smoking status, hypertension, diabetes, and FEV_1_ (%) predicted at visit

Figure S1. Flow chart of enrollment of COPDGene study population


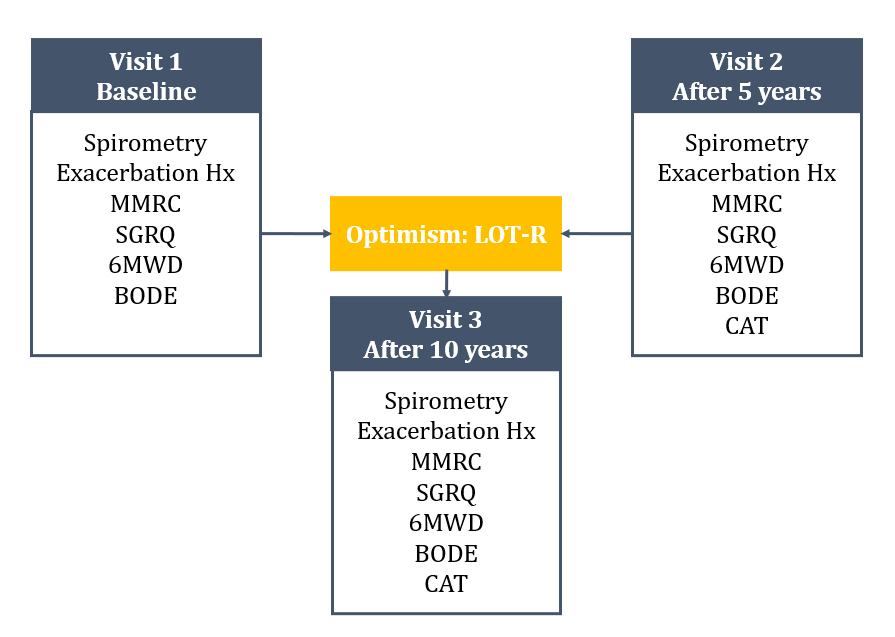


**COPDGene® Investigators – Core Units**

Administrative Center: James D. Crapo, MD (PI); Edwin K. Silverman, MD, PhD (PI); Barry J. Make, MD; Elizabeth A. Regan, MD, PhD

Genetic Analysis Center: Terri H. Beaty, PhD; Peter J. Castaldi, MD, MSc; Michael H. Cho, MD, MPH; Dawn L. DeMeo, MD, MPH; Adel El Boueiz, MD, MMSc; Marilyn G. Foreman, MD, MS; Auyon Ghosh, MD; Lystra P. Hayden, MD, MMSc; Craig P. Hersh, MD, MPH; Jacqueline Hetmanski, MS; Brian D. Hobbs, MD, MMSc; John E. Hokanson, MPH, PhD; Wonji Kim, PhD; Nan Laird, PhD; Christoph Lange, PhD; Sharon M. Lutz, PhD; Merry-Lynn McDonald, PhD; Dmitry Prokopenko, PhD; Matthew Moll, MD, MPH; Jarrett Morrow, PhD; Dandi Qiao, PhD; Elizabeth A. Regan, MD, PhD; Aabida Saferali, PhD; Phuwanat Sakornsakolpat, MD; Edwin K. Silverman, MD, PhD; Emily S. Wan, MD; Jeong Yun, MD, MPH

Imaging Center: Juan Pablo Centeno; Jean-Paul Charbonnier, PhD; Harvey O. Coxson, PhD; Craig J. Galban, PhD; MeiLan K. Han, MD, MS; Eric A. Hoffman, Stephen Humphries, PhD; Francine L. Jacobson, MD, MPH; Philip F. Judy, PhD; Ella A. Kazerooni, MD; Alex Kluiber; David A. Lynch, MB; Pietro Nardelli, PhD; John D. Newell, Jr., MD; Aleena Notary; Andrea Oh, MD; Elizabeth A. Regan, MD, PhD; James C. Ross, PhD; Raul San Jose Estepar, PhD; Joyce Schroeder, MD; Jered Sieren; Berend C. Stoel, PhD; Juerg Tschirren, PhD; Edwin Van Beek, MD, PhD; Bram van Ginneken, PhD; Eva van Rikxoort, PhD; Gonzalo Vegas SanchezFerrero, PhD; Lucas Veitel; George R. Washko, MD; Carla G. Wilson, MS;

PFT QA Center, Salt Lake City, UT: Robert Jensen, PhD

Data Coordinating Center and Biostatistics, National Jewish Health, Denver, CO: Douglas Everett, PhD; Jim Crooks, PhD; Katherine Pratte, PhD; Matt Strand, PhD; Carla G. Wilson, MS

Epidemiology Core, University of Colorado Anschutz Medical Campus, Aurora, CO: John E. Hokanson, MPH, PhD; Erin Austin, PhD; Gregory Kinney, MPH, PhD; Sharon M. Lutz, PhD; Kendra A. Young, PhD

Mortality Adjudication Core: Surya P. Bhatt, MD; Jessica Bon, MD; Alejandro A. Diaz, MD, MPH; MeiLan K. Han, MD, MS; Barry Make, MD; Susan Murray, ScD; Elizabeth Regan, MD; Xavier Soler, MD; Carla G. Wilson, MS

Biomarker Core: Russell P. Bowler, MD, PhD; Katerina Kechris, PhD; Farnoush Banaei-Kashani, PhD

**COPDGene® Investigators – Clinical Centers**

Ann Arbor VA: Jeffrey L. Curtis, MD; Perry G. Pernicano, MD

Baylor College of Medicine, Houston, TX: Nicola Hanania, MD, MS; Mustafa Atik, MD; Aladin Boriek, PhD; Kalpatha Guntupalli, MD; Elizabeth Guy, MD; Amit Parulekar, MD;

Brigham and Women’s Hospital, Boston, MA: Dawn L. DeMeo, MD, MPH; Craig Hersh, MD, MPH; Francine L. Jacobson, MD, MPH; George Washko, MD

Columbia University, New York, NY: R. Graham Barr, MD, DrPH; John Austin, MD; Belinda D’Souza, MD; Byron Thomashow, MD

Duke University Medical Center, Durham, NC: Neil MacIntyre, Jr., MD; H. Page McAdams, MD; Lacey Washington, MD

HealthPartners Research Institute, Minneapolis, MN: Charlene McEvoy, MD, MPH; Joseph Tashjian, MD

Johns Hopkins University, Baltimore, MD: Robert Wise, MD; Robert Brown, MD; Nadia N. Hansel, MD, MPH; Karen Horton, MD; Allison Lambert, MD, MHS; Nirupama Putcha, MD, MHS

Lundquist Institute for Biomedical Innovation at Harbor UCLA Medical Center, Torrance, CA: Richard Casaburi, PhD, MD; Alessandra Adami, PhD; Matthew Budoff, MD; Hans Fischer, MD; Janos Porszasz, MD, PhD; Harry Rossiter, PhD; William Stringer, MD

Michael E. DeBakey VAMC, Houston, TX: Amir Sharafkhaneh, MD, PhD; Charlie Lan, DO

Minneapolis VA: Christine Wendt, MD; Brian Bell, MD; Ken M. Kunisaki, MD, MS

Morehouse School of Medicine, Atlanta, GA: Eric L. Flenaugh, MD; Hirut Gebrekristos, PhD; Mario Ponce, MD; Silanath Terpenning, MD; Gloria Westney, MD, MS

National Jewish Health, Denver, CO: Russell Bowler, MD, PhD; David A. Lynch, MB

Reliant Medical Group, Worcester, MA: Richard Rosiello, MD; David Pace, MD

Temple University, Philadelphia, PA: Gerard Criner, MD; David Ciccolella, MD; Francis Cordova, MD; Chandra Dass, MD; Gilbert D’Alonzo, DO; Parag Desai, MD; Michael Jacobs, PharmD; Steven Kelsen, MD, PhD; Victor Kim, MD; A. James Mamary, MD; Nathaniel Marchetti, DO; Aditi Satti, MD; Kartik Shenoy, MD; Robert M. Steiner, MD; Alex Swift, MD; Irene Swift, MD; Maria Elena Vega-Sanchez, MD

University of Alabama, Birmingham, AL: Mark Dransfield, MD; William Bailey, MD; Surya P. Bhatt, MD; Anand Iyer, MD; Hrudaya Nath, MD; J. Michael Wells, MD

University of California, San Diego, CA: Douglas Conrad, MD; Xavier Soler, MD, PhD; Andrew Yen, MD

University of Iowa, Iowa City, IA: Alejandro P. Comellas, MD; Karin F. Hoth, PhD; John Newell, Jr., MD; Brad Thompson, MD

University of Michigan, Ann Arbor, MI: MeiLan K. Han, MD MS; Ella Kazerooni, MD MS; Wassim Labaki, MD MS; Craig Galban, PhD; Dharshan Vummidi, MD

University of Minnesota, Minneapolis, MN: Joanne Billings, MD; Abbie Begnaud, MD; Tadashi Allen, MD

University of Pittsburgh, Pittsburgh, PA: Frank Sciurba, MD; Jessica Bon, MD; Divay Chandra, MD, MSc; Joel Weissfeld, MD, MPH

University of Texas Health, San Antonio, San Antonio, TX: Antonio Anzueto, MD; Sandra Adams, MD; Diego Maselli-Caceres, MD; Mario E. Ruiz, MD; Harjinder Singh
